# Vaccination coverage and factors associated with incomplete polio vaccine schedule in children born in 2017-2018, in state capitals and interior region municipalities of Northeast Brazil

**DOI:** 10.1590/S2237-96222024v33e20231219.especial2.en

**Published:** 2025-01-10

**Authors:** Alberto Novaes Ramos, Ramon da Costa Saavedra, Adjoane Mauricio Silva Maciel, Anderson Fuentes Ferreira, Taynara Lais Silva, Luisa Helena de Oliveira Lima, Carla Magda Allan Santos Domingues, Jaqueline Caracas Barbosa, Ligia Regina Franco Sansigolo Kerr, Ana Paula França, Pallysson Paulo da Silva, Maria da Gloria Teixeira, José Cássio de Moraes, ICV 2020 Group, Adriana Ilha da Silva, Alberto Novaes Ramos, Ana Paula França, Andrea de Nazaré Marvão Oliveira, Antonio Fernando Boing, Carla Magda Allan Santos Domingues, Consuelo Silva de Oliveira, Ethel Leonor Noia Maciel, Ione Aquemi Guibu, Isabelle Ribeiro Barbosa Mirabal, Jaqueline Caracas Barbosa, Jaqueline Costa Lima, José Cássio de Moraes, Karin Regina Luhm, Karlla Antonieta Amorim Caetano, Luisa Helena de Oliveira Lima, Maria Bernadete de Cerqueira Antunes, Maria da Gloria Teixeira, Maria Denise de Castro Teixeira, Maria Fernanda de Sousa Oliveira Borges, Rejane Christine de Sousa Queiroz, Ricardo Queiroz Gurgel, Rita Barradas Barata, Roberta Nogueira Calandrini de Azevedo, Sandra Maria do Valle Leone de Oliveira, Sheila Araújo Teles, Silvana Granado Nogueira da Gama, Sotero Serrate Mengue, Taynãna César Simões, Valdir Nascimento, Wildo Navegantes de Araújo

**Affiliations:** 1 Universidade Federal do Ceará, Faculdade de Medicina, Fortaleza, CE, Brazil; 2 Universidade Federal do Ceará, Faculdade de Medicina, Fortaleza, CE, Brazil; 3 Universidade Federal da Bahia, Instituto de Saúde Coletiva, Salvador, BA, Brazil; 4 Secretaria Municipal de Saúde, Russas, CE, Brazil; 5 Universidade Federal do Piauí, Campus Senador Helvídio Nunes de Barros, Picos, PI, Brazil; 6 Organização Pan-Americana da Saúde, Brasília, DF, Brazil; 7 Faculdade de Ciências Médicas, Santa Casa de São Paulo, São Paulo, SP, Brazil; Universidade Federal do Espírito Santo, Vitória, ES, Brazil; Universidade Federal do Ceará, Departamento de Saúde Comunitária, Fortaleza, CE, Brazil; Faculdade Ciências Médicas Santa Casa de São Paulo, São Paulo, SP, Brazil; Secretaria de Estado da Saúde do Amapá, Macapá, AP, Brazil; Universidade Federal de Santa Catarina, SC, Brazil; Organização Pan-Americana da Saúde, Brasília, DF, Brazil; Instituto Evandro Chagas, Belém, PA, Brazil; Faculdade de Ciências Médicas Santa Casa de São Paulo, Departamento de Saúde Coletiva, São Paulo, SP, Brazil; Universidade Federal de Mato Grosso, Cuiabá, MT, Brazil; Universidade Federal do Paraná, Curitiba, PR, Brazil; Universidade Federal de Goiás, Goiânia, GO, Brazil; Universidade Federal do Piauí, Teresina, PI, Brazil; Universidade de Pernambuco, Faculdade de Ciências Médicas, Recife, PE, Brazil; Instituto de Saúde Coletiva, Universidade Federal da Bahia, Salvador, BA, Brazil; Secretaria de Estado da Saúde de Alagoas, Maceió, AL, Brazil; Universidade Federal do Acre, Rio Branco, AC, Brazil; Universidade Federal do Maranhão, Departamento de Saúde Pública, São Luís, MA, Brazil; Universidade Federal de Sergipe, Aracaju, SE, Brazil; Secretaria Municipal de Saúde, Boa Vista, RR, Brazil; Fundação Oswaldo Cruz, Mato Grosso do Sul, Campo Grande, MS, Brazil; Fundação Oswaldo Cruz, Escola Nacional de Saúde Pública Sergio Arouca, Rio de Janeiro, RJ, Brazil; Universidade Federal do Rio Grande do Sul, Porto Alegre, RS, Brazil; Fundação Oswaldo Cruz, Instituto de Pesquisa René Rachou, Belo Horizonte, MG, Brazil; Secretaria de Desenvolvimento Ambiental de Rondônia, Porto Velho, RO, Brazil; Universidade de Brasília, Brasília, DF, Brazil

**Keywords:** Cobertura Vacunal, Vacunas contra Poliovirus, Poliomielitis, Encuesta Epidemiológica, Vaccination Coverage, Poliovirus Vaccines, Poliomyelitis, Health Surveys

## Abstract

**Objective:**

To analyse vaccination coverage and factors associated with incomplete polio vaccination in a cohort of children born in 2017-2018, in state capitals and interior region municipalities of Northeast Brazil.

**Methods:**

Household survey of children aged ≤24 months conducted between 2020 and 2022. Vaccination coverage and dropout rates were estimated, as well as factors associated with incomplete vaccination, analyzed by calculating odds ratios (OR) and 95% confidence intervals (95%CI).

**Results:**

**:** Among 12,137 children, vaccination coverage (4 doses) was 80.9% (95%CI 78.4;83.1); 8.4% were not vaccinated. Not having a vaccination card (OR=18.06; 95%CI 10.01;32.61) and use of private services (OR=1.46; 95%CI 1.23;1.74) were associated with incomplete vaccination. Higher dropout rates were found for the booster dose, especially in the highest stratum.

**Conclusion:**

Low vaccination coverage, poor dose follow-up and high dropout rates were found for polio vaccines in the areas studied.

## Study contributions

**Main results:**

80.9% poliomyelitis vaccination coverage, with non-vaccination associated with both better and poorer sociodemographic conditions, in addition to use of private health services, having more than one child per mother and not having a vaccination card.

**Implications for services:**

High risk of poliomyelitis reemerging highlights the need to expand access to healthcare, with greater reinforcement of effective vaccination strategies. Actions must prioritize primary care in different territorial realities in Northeast Brazil.

**Perspectives:**

The evidence generated provides additional support for decision-making in the Brazilian National Health System based on microplanning, aiming to overcome critical barriers to access to health services, and thus achieve poliomyelitis vaccine coverage targets.

## RESUMEN

**Objetivo:**

Analizar la cobertura vacunal y factores asociados al esquema incompleto de vacunación contra la polio en una cohorte de nacidos vivos en capitales y municipios del interior del Nordeste de Brasil entre 2017-2018.

**Métodos:**

Encuesta domiciliaria a niños de ≤ 24 meses entre 2020-2022. Se estimaron cobertura de vacunación, tasas de abandono, así como factores asociados a vacunación incompleta, analizados mediante cálculo de odds ratios (OR) e intervalos de confianza del 95% (IC_95_%).

**Resultados:**

En 12.137 niños, la cobertura vacunal (4 dosis) fue del 80,9% (IC_95_% 78,4;83,1); el 8,4% no estaban vacunados. Residir en estrato socioeconómico más alto (OR=1,42; IC_95_% 1,23;1,65) y no disponer de carnet de vacunación (OR=14,05; IC_95_% 8,54;23,12) se asociaron a vacunación incompleta. Se observaron tasas de abandono más elevadas para dosis de refuerzo, especialmente en estrato superior.

**Conclusión:**

En zonas estudiadas se observaron baja cobertura, un seguimiento reducido de dosis y altas tasas de abandono de vacunas antipoliomielíticas.

## INTRODUCTION

Poliomyelitis is a vaccine-preventable highly infectious viral disease, mainly affecting children under 5 years old, and can cause permanent flaccid paralysis of limbs as well as death. Wild poliovirus transmission occurs via the feces of infected people, especially children, in contexts of high social vulnerability, such as poor basic sanitati^on.^ 1,2

In Brazil, control actions began in the 1960s, with the adoption of virus-blocking vaccinat^i^on^3^ in areas with outbreaks, moving on to specific mass vaccination campai^g^ns^3^, followed by systematic campaigns in the following deca^de.^ 2,4 During that period, more than 25,000 poliomyelitis cases were recorded in Brazil, with approximately one third of this total in the Northeast region of the count^r^y.^5^ Despite the 90% reduction in cases of the disease between 1980 and 19^8^1,^6^ the Northeast region experienced serious epidemics in the 1980s, indicating the need for actions to contain circulation of the vir^us.^ 7,8

The launch of the Global Polio Eradication Initiative in 1988 in Brazil led to the worldwide eradication of two of the three serotypes - wild poliovirus types PVS2 and PVS3. However, as at the end of 2022, endemic transmission of PVS1 persisted in Afghanistan and Pakist^a^n.^2^ Moreover, at the end of 2022, vaccine-derived poliovirus (VDPV) cases in unvaccinated people reemerged in Israel and in the United Stat^e^s.^2^

This more recent scenario has led to concerns regarding greater susceptibility to reemergence of the disease, particularly given the current context of operational shortcomings during the COVID-19 pandemic, when global childhood poliomyelitis vaccination coverage dropped between 5% and 81%, from 2020 to 2021, notwithstanding the improvement in 20^22.^ 2,3

In Brazil, it is noteworthy that, in 1989, there were ten deaths due to acute poliomyelitis, half of which occurred in the Northeast region of the count^r^y.^5^ After five years without any cases, only in 1994 were Brazil and the entire region of the Americas certified as areas free from circulation of wild poliovir^u^s,^4^ ratifying the impact of vaccination.

The National Immunization Program (*Programa Nacional de Imunizações* - PNI) recommends vaccination, routinely and in campaigns, with three doses of inactivated poliovirus vaccine (IPV), at 2, 4 and 6 months, and two more booster doses with bivalent oral polio vaccine (bOPV), at 15 months and 4 years of age, with a vaccination coverage target of 9^5^%.^9^ Despite all the achievements of the PNI, the drop in vaccination coverage parameters to levels below established targets is a challenge, mainly with effect from 20^15,^ 6,8 in an even more critical manner in North and Northeast Braz^i^l.^8^ In the ^2^1st century, the 95% coverage target proposed by the PNI for the third dose of IPV vaccine was achieved in the Northeast region, for 16 years consecutively, from 2000 (97.2%) to 2015 (110.4%), but showed a significant reduction in 2016 (81.6%). Since then, the target for this vaccine has not been achiev^e^d.^5^

Within this scenario, recent research highlights the imminent risk of reemergence of wild poliovirus in Brazil, if measures are not taken to achieve PNI recommended targe^ts.7,8^,^10^ Estimating poliovirus vaccine coverage in Brazil and understanding what its influence represents are, therefore, strategic actions for achieving the targets. In view of this, the objective of this study was to analyze poliovirus vaccine coverage and factors associated with non-vaccination among a cohort of children born in 2017-2018, living in state capitals and interior region municipalities in Northeast Brazil.

## METHODS

### Study design 

Household-based health survey of birth cohorts comprised of household clusters in census tracts that were selected according to socioeconomic strata. With the aim of verifying the children’s vaccination situation, from birth to 24 months of age, the study used data from the Survey of vaccination coverage in the capital cities of 26 Brazilian States, Federal District and 12 interior region municipalities among children born in 2017-2018 living in urban areas (*Inquérito de cobertura vacinal nas capitais de 26 Estados, no Distrito Federal e em 12 municípios do interior em crianças nascidas em 2017-2018 residentes em* área *urbana* ^).11^,^12^

### Background

The survey was conducted in the nine capital cities of Northeast Brazil, São Luís (Maranhão), Teresina (Piauí), Fortaleza (Ceará), Natal (Rio Grande do Norte), João Pessoa (Paraíba), Recife (Pernambuco), Maceió (Alagoas), Aracaju (Sergipe) and Salvador (Bahia); and in four municipalities in the interior region of Northeast Brazil: Vitória da Conquista (Bahia), Caruaru (Pernambuco), Sobral (Ceará) and Imperatriz (Maranhão). In 2022, Northeast Brazil had an estimated population of 54,657,621 inhabitants (3,635,333, or 6.7%, aged 0-4 years), spread over 1,552,175 km², with a population density of 35.21 inhab./km². Aracaju was the least populous capital (602,757 inhab.), while Fortaleza was the most populous (2,428,708 inhab.). Among the municipalities in the interior region, Caruaru had the largest population (378,048 inhab^.)^.^13^

### Population and data source

The data used to obtain the sample came from the Live Birth Information System (*Sistema de Informação de Nascidos Vivos* - SINASC), whereby the target population was based on the records of children born alive in 2017-2018 in the municipalities covered by the survey. Survey data collection was carried out from September 2020 to March 2022. Primary data was obtained directly from the children’s parents/guardians and from analysis of the children’s vaccination cards.

### Data collection and study variables 

A standardized instrument was administered with parents/guardians of the children during home visits to obtain information about family, maternal and child characteristics (Table 2). The children’s vaccination cards were photographed to record and evaluate the basic vaccination schedule, considering vaccines administered in private services (at least one dose^).^ 11

**Table 1:** Distribution of the absolute and relative frequency (%) of the sample studied, by socioeconomic strata and study sites (state capitals and interior region municipalities), Northeast Brazil, 2020-2021 (n = 12,137)

Variables/socioeconomic strata	A n (%)	B n (%)	C n (%)	D n (%)	Total n (%)
**Total**	2,701 (22.3)	3,118 (25.7)	3,145 (25.9)	3,173 (26.1)	12,137 (100.0)
**State capitals**					
São Luís	182 (6.7)	223 (7.2)	224 (7.1)	225 (7.1)	854 (7.0)
Teresina	227 (8.4)	225 (7.2)	222 (7.1)	225 (7.1)	899 (7.4)
Fortaleza	312 (11.6)	432 (13.9)	423 (13.4)	445 (14.0)	1,612 (13.3)
Natal	84 (3.1)	153 (4.9)	223 (7.1)	225 (7.1)	685 (5.6)
João Pessoa	226 (8.4)	225 (7.2)	226 (7.2)	227 (7.2)	904 (7.4)
Recife	330 (12.2)	447 (14.3)	462 (14.7)	450 (14.2)	1,689 (13.9)
Maceió	205 (7.6)	279 (8.9)	219 (7.0)	226 (7.1)	929 (7.7)
Aracaju	233 (8.6)	219 (7.0)	222 (7.1)	226 (7.1)	900 (7.4)
Salvador	450 (16.7)	456 (14.6)	456 (14.5)	456 (14.4)	1,818 (15.0)
**Interior region municipalities**					
Imperatriz	120 (4.4)	113 (3.6)	118 (3.8)	114 (3.6)	465 (3.8)
Sobral	103 (3.8)	119 (3.8)	120 (3.8)	123 (3.9)	465 (3.8)
Caruaru	113 (4.2)	114 (3.7)	116 (3.7)	119 (3.8)	462 (3.8)
Vitória da Conquista	116 (4.3)	113 (3.6)	114 (3.6)	112 (3.5)	455 (3.7)

**Table 2:** Family, maternal and child sociodemographic characteristics (%) and 95% confidence interval (95%CI) of children born in 2017 and 2018, by socioeconomic strata, in state capitals and interior region municipalities, Northeast Brazil, 2020-2022 (n = 12,137)

**Variables/socioeconomic strata**	**A**	**B**	**C**	**D**	**Total**
	% (95%CI)	% (95%CI)	% (95%CI)	% (95%CI)	% (95%CI)
**Family characteristics**					
*Bolsa* *Família* **Program (yes)**	7.9 (5.7;11.0)	20.1 (15.7;25.4)	37.8 (33.7;42.1)	49.9 (45.5;54.3)	36.0 (33.4;38.7)
**Monthly family income (BRL)**					
≤ 1000	5.7 (3.8;8.3)	19.1 (15.3;23.6)	37.3 (32.5;42.2)	55.5 (51.1;59.9)	38.0 (35.0;41.2)
1001-3000	12.1 (8.6;16.6)	30.8 (25.5;36.7)	44.1 (39.4;49.0)	35 (30.5;39.8)	32.5 (29.7;35.5)
3001-8000	27.5 (20.6;35.8)	24.6 (19.7;30.4)	14.8 (10.4;20.6)	3.4 (2.5;4.7)	12.9 (10.9;15.3)
≥ 8001	35.3 (26.3;45.5)	11.9 (6.0;22.2)	1.1 (0.7;1.8)	0.3 (0.0;0.9)	8.0 (5.9;10.8)
Unable to answer/did not answer	19.4 (11.1;31.9)	13.6 (8.3;21.5)	2.8 (1.9;4.1)	5.8 (3.6;9.3)	8.5 (6.3;11.4)
**Maternal characteristics**					
**Age group when child born (years)**					
< 20	1.0 (0.6;1.9)	1.1 (0.7;1.8)	2.5 (1.8;3.6)	4.5 (3.3;6.2)	3.0 (2.4;3.9)
20-34	44.8 (37.0;52.8)	50.0 (44.2;55.9)	67.9 (64.5;71.2)	65.1 (61.3;68.7)	60.2 (57.5;62.8)
≥ 35	53.9 (45.8;61.8)	48.3 (42.2;54.4)	29.3 (26.1;32.6)	30 (26.1;34.3)	36.4 (33.6;39.3)
Unable to answer/did not answer	0.3 (0.2;0.6)	0.6 (0.3;1.0)	0.3 (0.0;0.8)	0.4 (0.2;1.0)	0.4 (0.2;0.6)
**Schooling (years)**					
0-8	2.1 (1.3;3.1)	6.3 (4.6;8.7)	8.3 (6.5;10.5)	15.8 (13.4;18.6)	10.5 (9.3;11.9)
9-12	4.8 (2.9;7.7)	9.9 (7.5;12.9)	18.0 (14.8;21.8)	22.1 (19;25.6)	16.6 (14.8;18.5)
13-15	28.0 (21.9;35.1)	33.0 (26.4;40.2)	54.1 (50.3;57.9)	49.4 (45.2;53.5)	44.5 (41.7;47.4)
≥ 16	61.9 (54.2;69.0)	47.6 (38.7;56.6)	17.1 (14.1;20.6)	10.1 (5.5;18.0)	25.6 (22.0;29.7)
Unable to answer/did not answer	3.3 (1.6;7.1)	3.3 (1.8;5.8)	2.5 (1.6;3.8)	2.6 (1.8;3.7)	2.8 (2.2;3.6)
**Paid job (yes)**	68.6 (60.8;75.4)	56.2 (50.8;61.4)	43.0 (39.5;46.5)	36.4 (33.0;39.9)	46.0 (43.5;48.7)
**Number of children alive (average)**	1.9 (1.9;2.0)	2.0 (1.9;2.0)	2.0 (1.9;2.1)	2.2 (2.2;2.3)	2.0 (2.0;2.1)
**Child characteristics**					
**Child’s sex**					
Male	50.4 (43.0;57.7)	51.4 (46.6;56.1)	51.6 (47.5;55.7)	50.7 (47.8;53.6)	50.9 (48.8;53.1)
Female	49.6 (42.3;57.0)	48.6 (43.9;53.4)	48.4 (44.3;52.5)	49.3 (46.4;52.2)	49.1 (46.9;51.2)
**Has a vaccination card (yes)**	98.9 (95.9;99.7)	99.3 (98.7;99.6)	99.1 (98.2;99.6)	99.0 (97.2;99.6)	99.0 (98.3;99.4)
**Use of a private service for vaccination (yes)**	52.2 (43.0;61.3)	26.0 (19.1;34.3)	7.8 (6.0;10.1)	5.7 (2.2;13.8)	16.9 (13.6;20.8)
**Attends daycare/school (yes)**	48.7 (37.9;59.6)	37.6 (29.9;45.9)	34.4 (29.8;39.4)	31.1 (26.7;35.8)	35.7 (32.4;39.1)

### Sampling procedure

The research sampling plan provided for a study population size based on a population of 384,005 live births in 2017-2018 at the study sites.

The method for obtaining the estimated sample of children was structured in three stages. Stage A: division of the census tracts of the selected municipalities according to socioeconomic strata. These strata were defined based on data on average family income, the proportion of literate parents/guardians and the proportion of parents/guardians with income ≥ 20 minimum wages. Subsequently, the census tracts were grouped by clusters, in accordance with the definition of the four socioeconomic stra^ta^.^13^ In Stage B, the addresses of children registered as living in the census tracts were georeferenced, to form clusters with 56 or more children in each stratum. In Stage C, a variable number of households in each socioeconomic stratum were selected at random, to be visited during the field activities.

The sample was characterized according to socioeconomic strata (A, B, C and D), in which classification A corresponds to the stratum with the best income and schooling indicators for heads of household, while strata D refers to the stratum with the poorest socioeconomic indicators in the municipalities surveyed. Sampling weights were calculated for each child included in the survey, based on selection probability, adjusted for non-response and design effe^ct^.^11^

### Analysis

The proportion of children vaccinated against poliomyelitis (first, second and third doses, and first booster) was calculated considering the completeness of the vaccination schedule in accordance with the four doses recommended by the PNI up to the age of the population covered by this stu^dy.^ 5,9 We assessed the evolution of vaccination coverage of the four doses, compared with the sequence defined for the vaccination schedule.

For the purposes of sequential analysis, we dichotomized the vaccination status dependent variable, considering the four poliovirus vaccine doses: incomplete vaccination or full vaccination (reference group). The full poliovirus vaccination schedule provides for three doses in the first year of life and a booster dose in the second year of life.

In order to calculate vaccination coverage, the most recent validated doses of the full schedule were considered, in relation to the total number of live births. The dropout rates for the second and third doses and the first booster in relation to the first dose were calculated, based on the vaccination coverage of each dose according to socioeconomic strata, capital city, interior region city and overall dropout, as follows:

dropout rate = first dose coverage – second or third dose coverage or booster / first dose coverage

The validated doses were grouped together in three classes:

- Non-vaccination ‒ no record of doses (no doses administered), incomplete vaccination;
- Incomplete dose schedule (between one and three doses administered);
- Full vaccination ‒ full dose schedule (four doses administered).

The data were organized according to socioeconomic stratum, year of birth, state capitals and interior region cities.

Weighted estimates of vaccination coverage of the four doses of poliovirus vaccine and 95% confidence intervals (95%CI) were calculated for each dose and for the full schedule according to socioeconomic strata and municipalities. These estimates were calculated based on a p value < 0.^05^.^11^

The following variables were selected in order to assess factors associated with incomplete vaccination: *Bolsa* *Família* Program (yes, no), monthly family income (BRL) (≤ 1000, 1001-3000, 3001-8000, ≥ 8001, unable to answer/did not answer), age group, in years (< 20, 20-34, ≥ 35, unable to answer/did not answer), schooling, in years of study (0-8, 9-12, 13-15, ≥ 16, unable to answer/did not answer), paid job (yes, no), number of children alive (average), child’s sex (male, female), has a vaccination card (yes, no), use of a private service for vaccination (yes, no), attends daycare/school (yes, no).

The sampling weights were calculated in two stages: 1) basic sampling weights, represented by the inverse of the inclusion probabilities of interviewed households; and 2) weights calibrated by known population tota^ls^.^11^

The analysis of risk factors associated with incomplete vaccination (not receiving all doses) was performed using logistic regression models and magnitude of association was estimated by calculating the adjusted odds ratio (OR-a) and respective 95%CI, in multiple regression models. Variables that showed association with a p value < 0.20, in the simple logistic regression model, were included in the analysis model using the stepwise method, in order to investigate the independent effect of those variables together on the occurrence of incomplete vaccination. Presence of collinearity between model variables was analyzed by calculating the variance inflation factor, and variables with evidence of collinearity were excluded from the analysis. We used Stata version 17 for the statistical calculations.

### Ethical considerations

The study was approved by the Research Ethics Committee of the *Instituto de Saúde Coletiva da Universidade Federal da Bahia*, as per Opinion No. 3.366.818, on June 4, 2019, and Certificate of Submission for Ethical Appraisal (*Certificado* *de* *Apresentação* *de* *Apreciação* Ética - CAAE) No. 4306919.5.0000.5030; and by the Research Ethics Committee of the *Irmandade da Santa Casa de São Paulo*, as per Opinion No. 4.380.019, on November 4, 2020, and CAAE No. 39412020.0.0000.5479.

## RESULTS

The interviews involved a total of 12,137 children, mostly from socioeconomic strata D (n = 3,173, 26.1%) and C (n = 3,145, 25.9%), the majority living in the Bahia state capital of Salvador (n = 1,818, 15.0%) and in the interior region municipalities of Sobral and Imperatriz (n = 465, 3.8%) (Table 1). In the state capitals, an estimated loss of 525 children was originally expect^ed^.^11^

Being a *Bolsa* *Famíla* Program beneficiary was reported by 36.0% (95%CI 33.4;38.7) of parents/guardians, with greater frequency in stratum D (49.9%, 95%CI 45.5;54.3). 38.0% (95%CI 35.0;41.2) of the families had income of up to BRL 1,000.00. The majority of mothers were in the 20-34 age group (60.2%, 95%CI 57.5;62.8), had 13-15 years of schooling (44.5% 95%CI 41.7;47.4) and had a paid job (46.0%, 95%CI 43.5;48.7). The average number of children per mother was 2.0 (95%CI 2.0;2.1) (Table 2).

Among the children analyzed, there was a higher proportion with the following characteristics: males (50.9%, 95%CI 48.8;53.1) and having a vaccination card (99.0%, 95%CI 98.3;99.4). Use of a private vaccination service was stated for 16.9% (95%CI 13.6;20.8) of children, more frequently in stratum A (52.2%, 95%CI 43.0;61,3). Attending daycare/school was reported for 35.7% of the children (95%CI 32.4;39.1) (Table 2).

**Figure 1:**
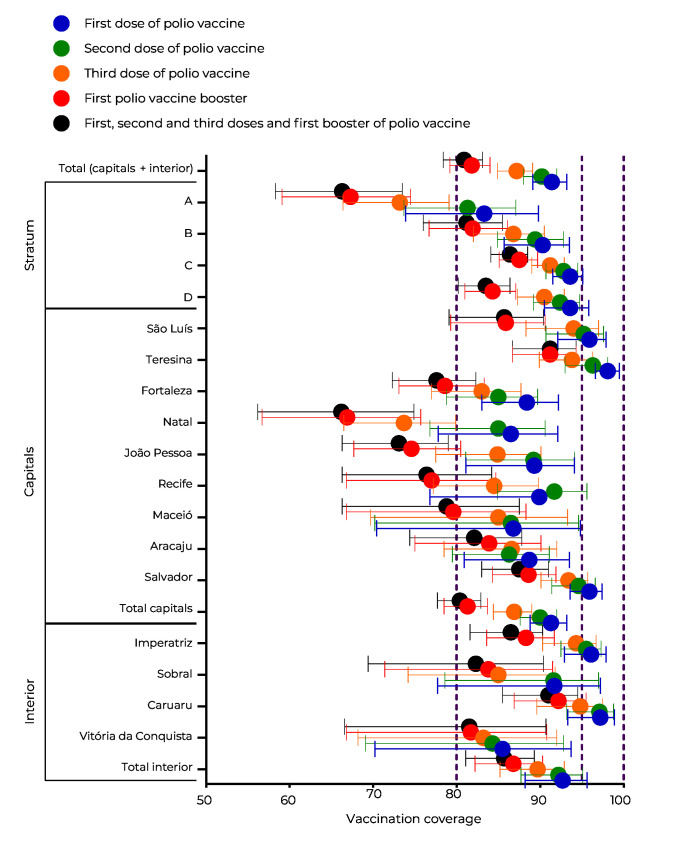
Vaccination coverage for first, second and third doses, and first booster, and full vaccination coverage against poliomyelitis in children born in 2017 and 2018, by socioeconomic strata and municipalities (state capitals and interior region municipalities), Northeast Brazil (n = 12,137)

Overall, full poliovirus vaccination coverage totaled 80.9% (95%CI 78.4;83.1), with a higher and lower proportion, respectively, in stratum C (86.4%, 95%CI 84.1;88.5) and stratum A (66.3%, 95%CI 58.3;73.5). The highest vaccination coverage was found for the first dose (91.4%, 95%CI 89.1;93.2), mainly in strata C and D (93.6%, 95%CI 91.5;95.1, 95%CI 90.5;95.8, respectively). The lowest vaccination coverage was found for th^e^ 1st booster (81.8%, 95%CI 79.2;84.0), mainly in stratum A (67.3%, 95%CI 59.1;74.5). The state capital city with the best vaccination coverage for full doses was Teresina (91.2%, 95%CI 86.7;94.3), while the lowest coverage was found in Natal (66.2%, 95%CI 56.2;74.9). With regard to interior region municipalities, Vitória da Conquista had the lowest vaccination coverage (81.5%, 95%CI 66.6;90.7) (Figure 1).

8.1% of the children had not received any poliovirus vaccine dose, and 11.0% had not completed the vaccination schedule.

The multiple regression analysis highlighted the following variables associated with incomplete poliovirus vaccination: not being a *Bolsa* *Família* Program beneficiary (OR-a 1.15, 95%CI 1.01;1.29), family income up to BRL 1,000.00 (OR-a 1.19, 95%CI 1.06;1.35), mother without a paid job (OR-a 1.21, 95%CI 1.08;1.35), more than one child per mother (OR-a 1.16, 95%CI 1.11;1.21), not having a vaccination card (OR-a 18.06, 95%CI 10.01;32.61) and use of a private vaccination service (OR-a 1.46, 95%CI 1.23;1.74) (Table 3).

**Table 3:** Crude and adjusted odds ratio (OR) and 95% confidence interval (95%CI), of association of sociodemographic factors with incomplete vaccination against poliomyelitis, in children born births in 2017 and 2018, residing in state capitals and interior region municipalities, Northeast Brazil, 2020-2022 (n = 12,137)

Characteristics	Crude OR (95%CI)	p-value	Adjusted OR (95%CI)	p-value
Socioeconomic stratum				
A	1.66 (1.46;1.89)	0.260	–	–
B	1.40 (1.23;1.59)		–	
C	1.00		–	
D	1.23 (1.08;1.4)		–	
**Municipality**				
Capital	1.00	0.679	–	–
Interior	1.03 (0.91;1.16)		–	
**Family characteristics**				
*Bolsa* *Família* **Program**				
Yes	1.00	< 0.001	1.00	0,143
No	1.20 (1.10;1.32)		1.15 (1.01;1.29)	
**Monthly family income (BRL)**				
≤ 1000	1.12 (1.00;1.25)	< 0.001	1.19 (1.06;1.35)	0,143
1001-3000	1.00		1,00	
3001-8000	1.12 (0.97;1.29)		0.94 (0.79;1.12)	
≥ 8001	1.74 (1.47;2.05)		1.21 (0.97;1.51)	
**Maternal characteristics**				
**Age group when child born (years)**				
< 20	1.00	0.243	–	–
20-34	1.30 (0.97;1.74)		–	
≥ 35	1.33 (0.99;1.79)		–	
**Schooling (years)**				
0-8	1.00	0.003	1.00	0.941
9-12	1.04 (0.88;1.23)		1.13 (0.94;1.36)	
13-15	0.91 (0.79;1.06)		1.04 (0.87;1.24)	
≥ 16 years	1.29 (1.10;1.51)		1.16 (0.93;1.47)	
**Paid job**				
Yes	1.00	< 0.001	1.00	0.003
No	1.29 (1.18;1.41)		1.21 (1.08;1.35)	
**Number of children alive**				
**Average of more than one child per mother**	1.10 (1.07;1.14)	< 0.001	1.16 (1.11;1.21)	< 0.001
**Child characteristics**				
**Sex**				
Male	1.00	0.995	–	–
Female	1.00 (0.91;1.09)		–	
**Has a vaccination card**				
Yes	1.00	< 0.001	1.00	< 0.001
No	13.01 (8.19;20.66)		18.06 (10.01;32.61)	
**Use of a private service for vaccination**				
Yes	1.69 (1.50;1.90)	< 0.001	1.46 (1.23;1.74)	< 0.001
No	1.00		1.00	
**Attends daycare/school**				
Yes	1.00	< 0.001	1.00	0.051
No	1.19 (1.09;1.31)		1.11 (1.00;1.23)	

Stratum A had a higher proportion of children with no record of administered doses (14.7%, 95%CI 9.5;22.0) and a higher proportion of children with an incomplete dose schedule (19.0%, 95%CI 13.6;25.9). Among the state capital cities, Natal/RN had the highest proportion of children with no recorded doses (13.3%, 95%CI 7.6;22.1). With regard to incomplete doses, the following state capitals stood out: Natal (20.5%, 95%CI 14.0;29.1); João Pessoa (17.3%, 95%CI 13.7;21.7); and Recife (16.1%, 95%CI 11.2;22.4). In the interior region cities, Vitória da Conquista had a higher proportion of children with no record of any administered dose of poliovirus vaccine (14.5%, 95%CI 6.3;29.8) (Table 4).

**Table 4:** Non-vaccination, incomplete vaccination, vaccination coverage against poliomyelitis and dropout rate (%) and 95% confidence interval (95%CI), in children born in 2017 and 2018, by socioeconomic stratum, state capitals and interior region municipalities, Northeast Brazil, 2020-2022 (n = 12,137)

Variable	Vaccination coverage			Dropout rate		
	Non-vaccination	Incomplete vaccination	Full vaccination	Second dose	Third dose	First booster
	% (95%CI)	% (95%CI)	% (95%CI)	%	%	%
Socioeconomic strata						
A	14.7 (9.5;22.0)	19.0 (13.6;25.9)	66.3 (58.3;73.5)	2.4	12.1	19.2
B	9.5 (6.3;14.1)	9.3 (6.8;12.6)	81.2 (76.0;85.5)	0.9	3.9	9.3
C	6.3 (4.8;8.4)	7.3 (5.9;8.9)	86.4 (84.1;88.5)	0.8	2.6	6.4
D	6.2 (4.0;9.3)	10.4 (8.7;12.3)	83.5 (80.2;86.4)	1.3	3.4	10.0
**State capitals**						
São Luís	4.0 (2.0;7.8)	10.3 (6.8;15.3)	85.7 (79.1;90.4)	0.7	2.0	10.5
Teresina	1.9 (1.1;3.4)	6.9 (4.3;10.9)	91.2 (86.7;94.3)	1.9	4.4	7.0
Fortaleza	11.6 (7.8;17.0)	10.7 (7.1;16.0)	77.6 (72.3;82.3)	3.8	6.1	11.0
Natal	13.3 (7.6;22.1)	20.5 (14.0;29.1)	66.2 (56.2;74.9)	1.8	14.7	22.7
João Pessoa	9.6 (4.9;18.0)	17.3 (13.7;21.7)	73.1 (66.3;79.0)	0.0	4.9	16.4
Recife	7.6 (3.8;14.4)	16.1 (11.2;22.4)	76.4 (66.3;84.2)	-2.0	6.0	14.4
Maceió	12.8 (4.9;29.6)	8.4 (5.4;12.9)	78.8 (66.3;87.5)	0.4	2.1	8.3
Aracaju	11.3 (6.4;19.1)	6.6 (4.2;10.2)	82.1 (74.4;87.8)	2.7	2.3	5.4
Salvador	3.8 (2.4;6.0)	8.7 (6.2;12.1)	87.5 (83.0;91.0)	1.4	2.6	7.6
State capitals	8.2 (6.4;10.4)	11.4 (9.8;13.2)	80.4 (77.7;82.9)	1.4	4.8	10.9
**Interior region municipalities**						
Imperatriz	3.7 (1.9;6.9)	9.8 (6.9;13.6)	86.5 (81.6;90.3)	0.6	1.9	8.2
Sobral	7.8 (2.6;21.0)	9.9 (6.1;15.7)	82.3 (69.4;90.4)	0.2	7.3	8.6
Caruaru	2.8 (1.1;6.7)	6.3 (3.6;10.7)	91.0 (85.5;94.5)	0.1	2.5	5.2
Vitória da Conquista	14.5 (6.3;29.8)	4.0 (2.2;7.4)	81.5 (66.6;90.7)	1.4	2.7	4.5
Interior	7.2 (4.3;11.7)	7.2 (5.5;9.3)	85.7 (81.1;89.3)	0.6	3.2	6.4
Overall	8.1 (6.5;10.1)	11.0 (9.6;12.7)	80.9 (78.4;83.1)	1.3	4.6	10.5

The dropout rate was higher for the first booster (10.5%), when compared to the first dose, with a smaller drop for the third dose (4.6%) compared to the first dose. Stratum A had the highest dropout rates for the first booster (20.0%) and the third dose (12.1%) compared to the first dose, being higher in Natal (27.1% dropout for the first booster in relation to the first dose; and 14.5% for the third dose, in relation to the first dose) and in Imperatriz (10.0% dropout for the first booster in relation to the first dose, and 2.7% for the third dose, in relation to the first dose) (Table 4).

## DISCUSSION

This study provided evidence of a critical situation regarding poliovirus vaccine coverage among children up to 24 months old, in state capitals and interior region cities of Northeast Brazil. It is noteworthy that the 95% the vaccination schedule target proposed by the PNI for children born alive in 2017-2018 was not achieved. Critically, almost one fifth of the child population analyzed had not been not fully vaccinated against poliomyelitis, and – most worryingly – 8.4% of the children in this study (more than 1,000 children) had no record of receiving any dose of vaccine against this potentially serious vaccine-preventable disease.

Non-vaccination and incomplete vaccination against poliomyelitis were jointly associated with factors that reflect part of the social inequities among children with low vaccination covera^ge^.^14^

The combination of this context of under-vaccination in Brazil and the weakening of surveillance actions regarding circulation of wild poliovirus, originating from persistently endemic areas worldwide, and VDPV, due to the continuous use of bOPV, in addition to migratory flo^ws^,^15^ increases the risk of reemergence of this disease, a real and critical threat due to the possibility of a Public Health Emergency of International Conce^rn.2,16^,^17^

In this aspect, successful control of poliomyelitis requires the incorporation of immunization strategies focused on providing quality vaccines and implementing campaigns, with broad governmental, scientific and community involvement through microplanning actions, with direct action in the territory that is being monitor^ed.15,18^,^19^

Considerable drops in vaccination coverage have occurred in all regions of Braz^i^l,^8^ with a continued downward trend in the last decade, in particular with effect from 2015-20^16^.^20^ This fact gained force during the COVID-19 pandemic due to the high dropout rate and heterogeneity in vaccination covera^ge,21^,^22^ increasing the risk of reemergence of poliomyelitis and other vaccine-preventable diseas^es.15,18^,^19^ In this context, sustained improvements in vaccination coverage are required as well as equitable access to vaccination, mainly to recover and surpass pre-pandemic vaccination leve^ls^.^23^

The decline in achieving the target proposed by the PNI is notable, in this study, when compared to the coverage of the initial and sequential doses of the poliovirus vaccine schedule. In this situation, Brazil is a country at very high r^i^sk^8^ of reemergence of poliomyelitis, with the worst situations recorded in the municipalities of Natal (state capital) and Vitória da Conquista (interior regio^n)^.^12^

Considering the extreme severity of this disease and the vulnerable contexts experienced in Northeast Brazil, an urgent call for rapid and assertive intervention, with strong participation from governments, the scientific community and the population, should be considered a priori^t^y.^8^

The mother/guardian not having the child’s vaccination card during the visit to the health center to vaccinate their child, proved to be an operational factor associated with incomplete poliovirus vaccination covera^ge^.^12^ It is noteworthy that this and other barriers to access to immunization actions require new strategies to achieve vaccination coverage within national and international targe^ts.1,^ 2,8

In addition to greater social vulnerability linked to lower coverage of poliovirus vaccines, we also found that living in socially and economically more favorable areas was associated with non-vaccination, this being a circumstance also found in other studies in Brazil, considering populations with greater internet acce^ss.^ 21 There is discussion as to whether greater access to social networks containing inadequate information, the strong spread of fake news and the dissemination of anti-vaccine discou^rs^e^24^ may have led to an increase in non-vaccination. Other studies in Brazil have demonstrated heterogeneity, with a better reach of vaccination coverage in areas of lower social vulnerabili^ty^.^20^

In the same context, children with lower coverage of poliovirus vaccines living in more favorable socioeconomic strata in the Brazilian state capitals and interior region cities surveyed had higher frequency of vaccination in private servic^es.^ 25 Conversely, children/families who use public health services had higher vaccination coverage, probably due to the link established with primary health care and, as such, keeping their vaccination schedule up to da^te.25^,^26^ The shortcomings in the interface between private and public health services in routine actions and national multivaccination campaigns, as well as the failure to report data on doses administer^ed^,^25^ are aspects that may have contributed and should be revisited.

The *Bolsa* *Família* Program brings into perspective the need for adequate vaccination follow-up, this being one of its requirements. Among a greater proportion of parents/guardians from poorer socioeconomic strata, the Program has been highlighted as a relevant government policy for vaccination adherence and, therefore, for achieving adequate poliovirus vaccination covera^ge^.^27^

Low income, not having a job and having more than one child per mother are issues that reflect social inequality, being highlighted in different Northeast Brazilian states as factors associated with non-vaccination, and bring into perspective contexts that increase health limitations, especially among childr^en^,^25^ due to restricted basic social righ^ts^.^13^

The imminent and constant risk of reemergence of poliomyelitis in Brazil, due to the current epidemiological and social scenario, indicates the need for strong actions to strengthen Brazilian National Health System vaccination programs in the Northeast region of the count^ry.28^,^29^

As such, there is a need to expand more effective strategies, considering the monitoring of unvaccinated children to maintain high and homogeneous vaccination cover^age15^,^16^ – the main barriers to access to vaccination –, greater availability of vaccination services and qualified health professionals to attend to them and reduce lost opportuniti^es.12,25^,^30^ There is a clear need to structure microplanning processes in primary health care territories, to gain a better understanding of the factors involv^ed^,^19^ in addition to ensuring political w^i^ll^8^ to implement measures to achieve homogeneous poliomyelitis vaccination coverage in the Northea^st^.^25^

The relevance of health professionals linked to vaccination acti^o^ns^6^ and the need to strengthen training and technical support for the operationalization of actions to implement adequate and effective vaccination strategies stand out, given the context of the local health situati^on^.^19^

There is also a need to strengthen surveillance actions. All countries must ensure high poliomyelitis vaccination coverage in their populations in order to achieve the target of global eradication, with interventions appropriate to national health systems. In addition to the routine actions to be intensified, environmental and wastewater surveillance must be implemented to allow early detection of “silent” poliovirus transmission in the population, moving forward beyond actions focused exclusively on clinical surveillance of acute flaccid paralysis only in case definiti^o^n.^2^

The limitations of this study are related to the method established for using data from the 2010 demographic census, in order to stratify census tracts and sample composition. With regard to the field research, limitations were due to difficulties in accessing families, because of the COVID-19 pandemic, and the restriction of researchers’ access to households, especially in the higher socioeconomic strata. The results may also have been influenced by the limited vaccination card image quality and the lack of standardization of records of doses and vaccines administered by public and private vaccination services. 

In conclusion, this study provides additional evidence about the low coverage of poliovirus vaccines, and the high percentage of non-vaccination against poliomyelitis among children up to 24 months of age living in state capitals and municipalities with large populations in the interior region of Northeast Brazil. These findings indicate the high risk of reemergence of poliomyelitis, a serious vaccine-preventable disease, providing support for decision-making in planning vaccination actions. Expanding access to primary health care, adopting strategies to achieve adequate poliomyelitis coverage for children in Northeast Brazil is recommended
