# Supplementary material for: Vaccination coverage and factors associated with incomplete polio vaccine schedule in children born in 2017-2018, in state capitals and interior region municipalities of Northeast Brazil
Source: Epidemiol Serv Saude. 2025 Jan 10;33(spe2):e20231219. doi: 10.1590/S2237-96222024v33e20231219.especial2.en (PMC11734596; doi:10.1590/S2237-96222024v33e20231219.especial2.en)
Supplement: Translation

# Translation: Cobertura vacinal e fatores associados ao esquema incompleto das vacinas contra poliomielite em nascidos vivos entre 2017 e 2018, em capitais e municípios do interior do Nordeste brasileiro

**DOI:** 10.1590/S2237-96222024v33e20231219.especial2.pt

**Authors:** Alberto Novaes Ramos, Ramon da Costa Saavedra, Adjoane Mauricio Silva Maciel, Anderson Fuentes Ferreira, Taynara Lais Silva, Luisa Helena de Oliveira Lima, Carla Magda Allan Santos Domingues, Jaqueline Caracas Barbosa, Ligia Regina Franco Sansigolo Kerr, Ana Paula França, Pallysson Paulo da Silva, Maria da Gloria Teixeira, José Cássio de Moraes, Grupo ICV 2020, Adriana Ilha da Silva, Alberto Novaes Ramos, Ana Paula França, Andrea de Nazaré Marvão Oliveira, Antonio Fernando Boing, Carla Magda Allan Santos Domingues, Consuelo Silva de Oliveira, Ethel Leonor Noia Maciel, Ione Aquemi Guibu, Isabelle Ribeiro Barbosa Mirabal, Jaqueline Caracas Barbosa, Jaqueline Costa Lima, José Cássio de Moraes, Karin Regina Luhm, Karlla Antonieta Amorim Caetano, Luisa Helena de Oliveira Lima, Maria Bernadete de Cerqueira Antunes, Maria da Gloria Teixeira, Maria Denise de Castro Teixeira, Maria Fernanda de Sousa Oliveira Borges, Rejane Christine de Sousa Queiroz, Ricardo Queiroz Gurgel, Rita Barradas Barata, Roberta Nogueira Calandrini de Azevedo, Sandra Maria do Valle Leone de Oliveira, Sheila Araújo Teles, Silvana Granado Nogueira da Gama, Sotero Serrate Mengue, Taynãna César Simões, Valdir Nascimento, Wildo Navegantes de Araújo

**Affiliations:** 1 Universidade Federal do Ceará, Faculdade de Medicina, Fortaleza, CE, Brasil; 2 Universidade Federal do Ceará, Faculdade de Medicina, Fortaleza, CE, Brasil; 3 Universidade Federal da Bahia, Instituto de Saúde Coletiva, Salvador, BA, Brasil; 4 Secretaria Municipal de Saúde, Russas, CE, Brasil; 5 Universidade Federal do Piauí, Campus Senador Helvídio Nunes de Barros, Picos, PI, Brasil; 6 Organização Pan-Americana da Saúde, Brasília, DF, Brasil; 7 Faculdade de Ciências Médicas, Santa Casa de São Paulo, São Paulo, SP, Brasil; Universidade Federal do Espírito Santo, Vitória, ES, Brasil; Universidade Federal do Ceará, Departamento de Saúde Comunitária, Fortaleza, CE, Brasil; Faculdade Ciências Médicas Santa Casa de São Paulo, São Paulo, SP, Brasil; Secretaria de Estado da Saúde do Amapá, Macapá, AP, Brasil; Universidade Federal de Santa Catarina, SC, Brasil; Organização Pan-Americana da Saúde, Brasília, DF, Brasil; Instituto Evandro Chagas, Belém, PA, Brasil; Faculdade de Ciências Médicas Santa Casa de São Paulo, Departamento de Saúde Coletiva, São Paulo, SP, Brasil; Universidade Federal de Mato Grosso, Cuiabá, MT, Brasil; Universidade Federal do Paraná, Curitiba, PR, Brasil; Universidade Federal de Goiás, Goiânia, GO, Brasil; Universidade Federal do Piauí, Teresina, PI, Brasil; Universidade de Pernambuco, Faculdade de Ciências Médicas, Recife, PE, Brasil; Instituto de Saúde Coletiva, Universidade Federal da Bahia, Salvador, BA, Brasil; Secretaria de Estado da Saúde de Alagoas, Maceió, AL, Brasil; Universidade Federal do Acre, Rio Branco, AC, Brasil; Universidade Federal do Maranhão, Departamento de Saúde Pública, São Luís, MA, Brasil; Universidade Federal de Sergipe, Aracaju, SE, Brasil; Secretaria Municipal de Saúde, Boa Vista, RR, Brasil; Fundação Oswaldo Cruz, Mato Grosso do Sul, Campo Grande, MS, Brasil; Fundação Oswaldo Cruz, Escola Nacional de Saúde Pública Sergio Arouca, Rio de Janeiro, RJ, Brasil; Universidade Federal do Rio Grande do Sul, Porto Alegre, RS, Brasil; Fundação Oswaldo Cruz, Instituto de Pesquisa René Rachou, Belo Horizonte, MG, Brasil; Secretaria de Desenvolvimento Ambiental de Rondônia, Porto Velho, RO, Brasil; Universidade de Brasília, Brasília, DF, Brasil

**Keywords:** Cobertura Vacinal, Vacinas contra Poliovírus, Poliomielite, Inquéritos Epidemiológicos

## Abstract

**Objetivo:**

Analisar a cobertura vacinal e os fatores associados ao esquema incompleto das vacinas contra a poliomielite em coorte de nascidos vivos de 2017-2018, em capitais e municípios do interior do Nordeste brasileiro.

**Métodos:**

Inquérito domiciliar em crianças com idade ≤ 24 meses, entre 2020 e 2022. Estimaram-se a cobertura vacinal, a taxa de abandono e fatores associados ao esquema de vacinação incompleto, analisados pela razão de chances (*odds ratio*, OR) e intervalos de confiança de 95% (IC_95%_).

**Resultados:**

Em 12.137 crianças, a cobertura vacinal (quatro doses) foi de 80,9% (IC_95%_ 78,4;83,1); 8,4% não eram vacinadas. Não possuir caderneta de vacinação (OR = 14,05; IC_95%_ 8,54;23,12) e uso de serviço privado (OR = 1,46; IC_95%_ 1,23;1,74) estiveram associados à vacinação incompleta. Taxas de abandono mais altas foram observadas na dose de reforço, principalmente no estrato alto.

**Conclusão:**

Observaram-se baixas coberturas, redução no seguimento das doses e elevadas taxas de abandono para vacinas contra a poliomielite nas áreas estudadas.

## Contribuições do estudo

**Principais resultados:**

Cobertura vacinal de 80,9% para poliomielite, com a não vacinação associada tanto a melhores quanto a piores condições sociodemográficas, além de uso de serviço privado de saúde, ter mais de um filho por mãe e ausência de caderneta de vacinação.

**Implicações para os serviços:**

O elevado risco de reintrodução da poliomielite reforça a necessidade de ampliar o acesso à saúde, com maior reforço de estratégias efetivas para vacinação. Ações devem priorizar a atenção primária em diferentes realidades territoriais da região Nordeste.

**Perspectivas:**

As evidências geradas dão suporte adicional à tomada de decisões no Sistema Único de Saúde fundamentadas no microplanejamento, visando superar barreiras críticas para o acesso à saúde, e assim se alcançarem as metas de coberturas das vacinas contra a poliomielite.

## INTRODUÇÃO

A poliomielite é uma doença viral imunoprevenível, com elevada infectividade, acometendo principalmente crianças menores de 5 anos de idade, podendo causar paralisia flácida permanente de membros e óbito. A transmissão do poliovírus selvagem ocorre pelas fezes de pessoas infectadas, especialmente crianças, em contextos com alta vulnerabilidade social, como precariedade de saneamento bási^co.^ 1,2

No Brasil, as ações de controle foram iniciadas na década de 1960, com a adoção de bloqueios vacin^a^is^3^ em áreas com ocorrência de surtos, evoluindo para campanhas pontuais de vacinação em ma^s^sa^4^ e, na década posterior, com campanhas sistemátic^as.^ 2,4 Nesse período, foram registrados mais de 25 mil casos de poliomielite no país, aproximadamente um terço desse total na região Nordes^t^e.^5^ Apesar da redução de 90% dos casos dessa doença entre 1980 e 19^8^1,^6^ a região apresentou epidemias graves nos anos 1980, indicando a necessidade de ações para a contenção da circulação vir^al.^ 7,8

O lançamento da Iniciativa Global de Erradicação da Poliomielite, em 1988, no Brasil, levou à erradicação mundial de dois dos três sorotipos ‒ poliovírus selvagem PVS2 e PVS3. No entanto, até o final de 2022, a transmissão endêmica de PVS1 persistia no Afeganistão e no Paquist^ã^o.^2^ Registrou-se, ainda, no final de 2022, o ressurgimento de casos por poliovírus derivado vacinal (PVDV) em pessoas não vacinadas em Israel e nos Estados Unid^o^s.^2^

Este cenário mais atual tem gerado preocupações em face da maior suscetibilidade ao ressurgimento da doença, particularmente diante do contexto presente de falhas operacionais após a pandemia de covid-19, quando a cobertura vacinal infantil mundial para poliomielite teve quedas percentuais entre 5% e 81%, de 2020 para 2021, apesar da melhora em 20^22.^ 2,3

No Brasil, ressalta-se que, em 1989, houve a ocorrência de dez óbitos por poliomielite aguda, sendo a metade na região Nordes^t^e.^5^ Após cinco anos sem ocorrências de casos, somente em 1994, o país e toda a região das Américas foram certificados como áreas livres de circulação do poliovírus selvag^e^m,^4^ ratificando o impacto da vacinação.

O Programa Nacional de Imunizações (PNI) recomenda a vacinação, de rotina e em campanhas, com três doses da vacina inativada poliomielite (VIP), aos 2, 4 e 6 meses de vida, e mais duas doses de reforço com a vacina oral poliomielite bivalente (VOPb), aos 15 meses e aos 4 anos de idade, com meta de cobertura vacinal de 9^5^%.^9^ Apesar de todas as conquistas do PNI, o declínio nos parâmetros de cobertura vacinal para níveis abaixo das metas estabelecidas mantém os desafios, principalmente a partir de 20^15,^ 6,8 de forma mais crítica nas regiões Norte e Nordes^t^e.^8^ No século XXI, a meta de 95% de cobertura proposta pelo PNI para a terceira dose da vacina VIP foi alcançada na região Nordeste, por 16 anos consecutivos, de 2000 (97,2%) a 2015 (110,4%), mas apresentou redução significativa em 2016 (81,6%). Desde então, não se alcançou mais a meta dessa vacina nos anos subsequent^e^s.^5^

Nesse cenário, pesquisas recentes ressaltam o risco iminente de reintrodução do poliovírus selvagem no país, se medidas não forem adotadas para alcance das metas preconizadas pelo P^NI.7,8^,^10^ Estimar a cobertura das vacinas contra a poliomielite no país e compreender o que a influência representa, portanto, são ações estratégicas com vistas ao alcance das metas estabelecidas. Nessa perspectiva, o objetivo deste estudo é analisar a cobertura das vacinas contra a poliomielite e os fatores associados à não vacinação em uma coorte de nascidos vivos de 2017-2018, residentes em capitais e municípios do interior do Nordeste brasileiro.

## MÉTODOS

### Desenho do estudo

Inquérito epidemiológico, de base domiciliar, em coortes de nascimentos estruturadas em conglomerados domiciliares de setores censitários que foram selecionados segundo estratos socioeconômicos. Visando à verificação do percurso vacinal da criança, desde o nascimento até 24 meses de idade, o estudo teve como referência os dados do *Inquérito de cobertura vacinal nas capitais de 26 Estados, no Distrito Federal e em 12 municípios do interior em crianças nascidas em 2017-2018 residentes em área urban* ^a.11^,^12^

### Contexto

A pesquisa foi realizada nas nove capitais dos estados da região Nordeste, São Luís (Maranhão), Teresina (Piauí), Fortaleza (Ceará), Natal (Rio Grande do Norte), João Pessoa (Paraíba), Recife (Pernambuco), Maceió (Alagoas), Aracaju (Sergipe) e Salvador (Bahia); e em quatro municípios no interior da mesma região: Vitória da Conquista (Bahia), Caruaru (Pernambuco), Sobral (Ceará) e Imperatriz (Maranhão). A região Nordeste tinha uma população estimada de 54.657.621 habitantes em 2022 (3.635.333, ou 6,7%, com idade de 0 a 4 anos), distribuída em 1.552.175 km², com densidade demográfica de 35,21 hab./km². Aracaju era a capital menos populosa (602.757 hab.), e Fortaleza, a mais populosa (2.428.708 hab.). Entre os municípios do interior, Caruaru apresentava a maior população (378.048 hab^.)^.^13^

### População e fonte de dados

Os dados para a obtenção da amostra foram oriundos do Sistema de Informação de Nascidos Vivos (Sinasc), tendo como população-alvo os registros de crianças nascidas vivas em 2017-2018 nos municípios pesquisados. A coleta de dados do inquérito foi realizada no período de setembro de 2020 a março de 2022. Os dados primários foram obtidos diretamente das pessoas responsáveis pela criança e a partir da análise dos cartões de vacinação.

### Coleta de dados e variáveis do estudo

Um instrumento padronizado foi aplicado a pais/responsáveis pelas crianças durante as visitas domiciliares para a obtenção de informações sobre as características familiares, maternas e da criança (Tabela 2). O cartão de vacinação das crianças foi fotografado para registro e avaliação do esquema vacinal básico, considerando-se vacinas aplicadas nos serviços privados (pelo menos uma dos^e)^.^11^

**Tabela 1:** Distribuição de frequência absoluta e relativa (%) da amostra estudada, segundo estratos socioeconômicos e locais de estudo (capitais e municípios do interior) da região Nordeste do Brasil, 2020-2021 (n = 12.137)

Variáveis/estratos socioeconômicos	A n (%)	B n (%)	C n (%)	D n (%)	Total n (%)
**Total**	2.701 (22,3)	3.118 (25,7)	3.145 (25,9)	3.173 (26,1)	12.137 (100,0)
**Capitais**					
São Luís	182 (6,7)	223 (7,2)	224 (7,1)	225 (7,1)	854 (7,0)
Teresina	227 (8,4)	225 (7,2)	222 (7,1)	225 (7,1)	899 (7,4)
Fortaleza	312 (11,6)	432 (13,9)	423 (13,4)	445 (14,0)	1.612 (13,3)
Natal	84 (3,1)	153 (4,9)	223 (7,1)	225 (7,1)	685 (5,6)
João Pessoa	226 (8,4)	225 (7,2)	226 (7,2)	227 (7,2)	904 (7,4)
Recife	330 (12,2)	447 (14,3)	462 (14,7)	450 (14,2)	1.689 (13,9)
Maceió	205 (7,6)	279 (8,9)	219 (7,0)	226 (7,1)	929 (7,7)
Aracaju	233 (8,6)	219 (7,0)	222 (7,1)	226 (7,1)	900 (7,4)
Salvador	450 (16,7)	456 (14,6)	456 (14,5)	456 (14,4)	1.818 (15,0)
**Município do interior**					
Imperatriz	120 (4,4)	113 (3,6)	118 (3,8)	114 (3,6)	465 (3,8)
Sobral	103 (3,8)	119 (3,8)	120 (3,8)	123 (3,9)	465 (3,8)
Caruaru	113 (4,2)	114 (3,7)	116 (3,7)	119 (3,8)	462 (3,8)
Vitória da Conquista	116 (4,3)	113 (3,6)	114 (3,6)	112 (3,5)	455 (3,7)

**Tabela 2:** Características sociodemográficas (%) e intervalo de confiança de 95% (IC_95_) da família, da mãe e da criança em nascidos de 2017 e 2018, segundo estratos socioeconômicos, em capitais e municípios do interior, Nordeste do Brasil, 2020-2022 (n 12.137)

Variáveis/estratos	A	B	C	D	Total
	% (IC_95_%)	% (IC_95_%)	% (IC_95_%)	% (IC_95_%)	% (IC_95_%)
**Características da família**					
**Programa Bolsa Família (sim)**	7,9 (5,7;11,0)	20,1 (15,7;25,4)	37,8 (33,7;42,1)	49,9 (45,5;54,3)	36,0 (33,4;38,7)
**Renda familiar mensal (R$)**					
≤ 1.000,00	5,7 (3,8;8,3)	19,1 (15,3;23,6)	37,3 (32,5;42,2)	55,5 (51,1;59,9)	38,0 (35,0;41,2)
1.001,00-3.000,00	12,1 (8,6;16,6)	30,8 (25,5;36,7)	44,1 (39,4;49,0)	35 (30,5;39,8)	32,5 (29,7;35,5)
3.001,00-8.000,00	27,5 (20,6;35,8)	24,6 (19,7;30,4)	14,8 (10,4;20,6)	3,4 (2,5;4,7)	12,9 (10,9;15,3)
≥ 8.001,00	35,3 (26,3;45,5)	11,9 (6,0;22,2)	1,1 (0,7;1,8)	0,3 (0,0;0,9)	8,0 (5,9;10,8)
Não sabe/não respondeu	19,4 (11,1;31,9)	13,6 (8,3;21,5)	2,8 (1,9;4,1)	5,8 (3,6;9,3)	8,5 (6,3;11,4)
**Características da mãe**					
**Faixa etária ao nascimento da criança (anos)**					
< 20	1,0 (0,6;1,9)	1,1 (0,7;1,8)	2,5 (1,8;3,6)	4,5 (3,3;6,2)	3,0 (2,4;3,9)
20-34	44,8 (37,0;52,8)	50,0 (44,2;55,9)	67,9 (64,5;71,2)	65,1 (61,3;68,7)	60,2 (57,5;62,8)
≥ 35	53,9 (45,8;61,8)	48,3 (42,2;54,4)	29,3 (26,1;32,6)	30 (26,1;34,3)	36,4 (33,6;39,3)
Não sabe/não respondeu	0,3 (0,2;0,6)	0,6 (0,3;1,0)	0,3 (0,0;0,8)	0,4 (0,2;1,0)	0,4 (0,2;0,6)
**Escolaridade (anos)**					
0-8	2,1 (1,3;3,1)	6,3 (4,6;8,7)	8,3 (6,5;10,5)	15,8 (13,4;18,6)	10,5 (9,3;11,9)
9-12	4,8 (2,9;7,7)	9,9 (7,5;12,9)	18,0 (14,8;21,8)	22,1 (19;25,6)	16,6 (14,8;18,5)
13-15	28,0 (21,9;35,1)	33,0 (26,4;40,2)	54,1 (50,3;57,9)	49,4 (45,2;53,5)	44,5 (41,7;47,4)
≥ 16	61,9 (54,2;69,0)	47,6 (38,7;56,6)	17,1 (14,1;20,6)	10,1 (5,5;18,0)	25,6 (22,0;29,7)
Não sabe/não respondeu	3,3 (1,6;7,1)	3,3 (1,8;5,8)	2,5 (1,6;3,8)	2,6 (1,8;3,7)	2,8 (2,2;3,6)
**Trabalho remunerado (sim)**	68,6 (60,8;75,4)	56,2 (50,8;61,4)	43,0 (39,5;46,5)	36,4 (33,0;39,9)	46,0 (43,5;48,7)
**Número de filhos vivos (média)**	1,9 (1,9;2,0)	2,0 (1,9;2,0)	2,0 (1,9;2,1)	2,2 (2,2;2,3)	2,0 (2,0;2,1)
**Características da criança**					
**Sexo da criança**					
Masculino	50,4 (43,0;57,7)	51,4 (46,6;56,1)	51,6 (47,5;55,7)	50,7 (47,8;53,6)	50,9 (48,8;53,1)
Feminino	49,6 (42,3;57,0)	48,6 (43,9;53,4)	48,4 (44,3;52,5)	49,3 (46,4;52,2)	49,1 (46,9;51,2)
**Possui caderneta (sim)**	98,9 (95,9;99,7)	99,3 (98,7;99,6)	99,1 (98,2;99,6)	99,0 (97,2;99,6)	99,0 (98,3;99,4)
**Uso de serviço privado para a vacinação (sim)**	52,2 (43,0;61,3)	26,0 (19,1;34,3)	7,8 (6,0;10,1)	5,7 (2,2;13,8)	16,9 (13,6;20,8)
**Frequenta creche/escola (sim)**	48,7 (37,9;59,6)	37,6 (29,9;45,9)	34,4 (29,8;39,4)	31,1 (26,7;35,8)	35,7 (32,4;39,1)

### Procedimento amostral

A partir do plano amostral da pesquisa, o tamanho da população do estudo foi baseado em uma população de 384.005 crianças nascidas vivas em 2017-2018 nos locais do estudo.

O método para a obtenção da amostra estimada das crianças foi estruturado em três etapas. Etapa A: divisão dos territórios dos municípios selecionados segundo estratos socioeconômicos. Esses estratos foram definidos com base em dados de renda média das pessoas responsáveis pelo domicílio, na proporção de pessoas responsáveis alfabetizadas e na proporção de responsáveis com renda ≥ 20 salários mínimos. Posteriormente, os setores foram agrupados por *clusters*, ajustando-se para a definição de quatro estrat^os^.^13^ Na Etapa B, realizou-se o georreferenciamento dos endereços das crianças nascidas vivas nos setores censitários, para serem compostos conglomerados com 56 ou mais crianças em cada estrato. Na Etapa C, selecionou-se por sorteio um número variável de residências em cada estrato socioeconômico, a fim de se orientarem atividades de campo.

A amostra foi caracterizada conforme estratos socioeconômicos (A, B, C e D), em que a classificação A corresponde ao estrato com melhores indicadores de renda e escolaridade dos chefes de família, e D, àquele com os piores indicadores nos municípios de residência do estudo. Para cada criança incluída na pesquisa, foram calculados os pesos da amostra, com base na probabilidade de seleção, ajustados para a não resposta e efeito do proje^to^.^11^

### Análise

A proporção de crianças vacinadas contra a poliomielite (primeira, segunda e terceira doses, e primeiro reforço) foi calculada considerando-se a completude do esquema com as quatro doses preconizadas pelo PNI até a idade da população deste estu^do.^ 5,9 Avaliou-se a evolução da cobertura vacinal dessas quatro doses, observando-se a sequência do calendário vacinal.

Para análise sequencial, a variável dependente do *status* de vacinação, considerando-se as quatro doses da vacina contra a poliomielite, foi dicotomizada: sem vacinação completa (incompletude vacinal) ou vacinação completa (grupo de referência). O esquema completo de vacinação para a poliomielite corresponde a três doses no primeiro ano de vida e a uma dose de reforço no segundo ano de vida.

Para o cálculo da cobertura vacinal, foram consideradas as últimas doses válidas do esquema completo, em relação ao total de nascidos vivos. Foram calculadas as taxas de abandono da segunda e terceira doses e do primeiro reforço em relação à primeira dose, com base nas coberturas vacinais de cada dose segundo estratos socioeconômicos, capitais, interior e geral, do seguinte modo:

taxa de abandono = cobertura de primeira dose – cobertura de segunda ou terceira doses ou reforço/cobertura de primeira dose

As doses válidas foram combinadas em três classes a seguir:

- Não vacinação ‒ sem registro de doses (não tomou nenhuma dose), vacinação incompleta;
- Esquema incompleto de doses (tomou de uma a três doses);
- Vacinação completa ‒ esquema completo de doses (tomou quatro doses).

Os dados foram organizados por estrato, ano de nascimento, capitais e municípios do interior.

Estimativas ponderadas de cobertura vacinal das quatro doses das vacinas da poliomielite e intervalos de confiança de 95% (IC_95%_) foram obtidos para cada dose e para o esquema completo segundo os estratos socioeconômicos e municípios. Essas estimativas foram geradas com base em um p-valor < 0,^05^.^11^

Para avaliação dos fatores associados à vacinação incompleta, foram selecionadas as seguintes variáveis: Programa Bolsa Família (PBF) (sim, não), renda familiar mensal (R$) (≤ 1.000,00, 1.001,00 a 3.000,00, 3.001,00 a 8.000,00, ≥ 8.001,00, não sabe/não respondeu), faixa etária, em anos (< 20, 20 a 34, ≥ 35, não sabe/não respondeu), escolaridade, em anos de estudo (0 a 8, 9 a 12, 13 a 15, ≥ 16, não sabe/não respondeu), trabalho remunerado (sim, não), número de filhos vivos (média), sexo da criança (masculino, feminino), possui caderneta (sim, não), uso de serviço privado para a vacinação (sim, não), frequenta creche/escola (sim, não).

Os pesos amostrais foram calculados em duas etapas: 1) pesos amostrais básicos, representados pelo inverso de probabilidades de inclusão de domicílios entrevistados; e 2) pesos calibrados pelos totais da população conheci^da^.^11^

A análise dos fatores de risco associados à vacinação incompleta (não receber todas as doses) foi realizada em modelos de regressão logística e a magnitude da associação estimada pelo cálculo do *odds ratio* ajustado (OR-a) e respectivos IC_95%_, em modelos de regressão múltipla. Variáveis analisadas que evidenciaram associação com p-valor < 0,20, no modelo de regressão logística simples, foram incluídas no modelo de análise pelo método *stepwise*, para se investigar o efeito independente dessas variáveis conjuntamente na ocorrência de vacinação incompleta. A verificação da presença de colinearidade entre variáveis do modelo foi analisada pelo cálculo do fator de inflação da variância, sendo que variáveis com evidência de colinearidade foram excluídas da análise. Foi utilizado o *software* Stata versão 17 para os cálculos estatísticos.

### Considerações éticas

A pesquisa foi aprovada pelos Comitês de Ética em Pesquisa com Seres Humanos do Instituto de Saúde Coletiva da Universidade Federal da Bahia, sob parece^r^ no 3.366.818, em 4 de junho de 2019, com Certificado de Apresentação de Apreciação Ética (CAAE) 4306919.5.0000.5030; e da Irmandade da Santa Casa de São Paulo, sob parece^r^ no 4.380.019, em 4 de novembro de 2020, com CAAE 39412020.0.0000.5479. 

## RESULTADOS

Foram incluídas 12.137 crianças, majoritariamente dos estratos socioeconômicos D (n = 3.173, 26,1%) e C (n = 3.145, 25,9%), e residentes na capital Salvador (n = 1.818, 15,0%) e nos municípios de interior de Sobral e Imperatriz (n = 465, 3,8%) (Tabela 1). Nas capitais, estimou-se perda de 525 crianças nascidas vivas previstas originalmen^te^.^11^

Ser beneficiário do PBF foi reportado por 36,0% (IC_95%_ 33,4;38,7) dos pais/responsáveis, com maior frequência no estrato D (49,9%, IC_95%_ 45,5;54,3). A renda foi de até R$ 1.000,00 em 38,0% (IC_95%_ 35,0;41,2) das famílias, a maior parte das mães encontrava-se na faixa etária de 20 a 34 anos (60,2%, IC_95%_ 57,5;62,8), possuía de 13 a 15 anos de estudo (44,5% IC_95%_ 41,7;47,4) e estava em trabalho remunerado (46,0%, IC_95%_ 43,5;48,7). A média de filhos por mãe foi de 2,0 (IC_95%_ 2,0;2,1) (Tabela 2).

Entre as crianças analisadas, verificou-se maior proporção com as seguintes características: sexo masculino (50,9%, IC_95%_ 48,8;53,1) e presença de caderneta de vacinação (99,0%, IC_95%_ 98,3;99,4). O uso de serviço privado de vacinação foi mencionado para 16,9% (IC_95%_ 13,6;20,8) das crianças, com maior frequência no estrato A (52,2%, IC_95%_ 43,0;61,3). Estar em creche/escola foi relatado para 35,7% das crianças (IC_95%_ 32,4;39,1) (Tabela 2).

A cobertura vacinal completa das vacinas da poliomielite foi de 80,9% (IC_95%_ 78,4;83,1), com maior e menor proporção, respectivamente, nos estratos C (86,4%, IC_95%_ 84,1;88,5) e A (66,3%, IC_95%_ 58,3;73,5). A maior cobertura vacinal foi verificada para a primeira dose (91,4%, IC_95%_ 89,1;93,2), principalmente nos estratos C e D (93,6%, IC_95%_ 91,5;95,1, IC_95%_ 90,5;95,8, respectivamente). A menor cobertura vacinal foi evidenciada para o 1º reforço (81,8%, IC_95%_ 79,2;84,0), principalmente no estrato A (67,3%, IC_95%_ 59,1;74,5). A capital com a melhor cobertura vacinal para doses completas foi Teresina (91,2%, IC_95%_ 86,7;94,3), enquanto a mais baixa foi observada em Natal (66,2%, IC_95%_ 56,2;74,9). Para os municípios do interior, Vitória da Conquista apresentou a menor cobertura vacinal (81,5%, IC_95%_ 66,6;90,7) (Figura 1).

**Figura 1:** Cobertura vacinal de primeira, segunda e terceira doses, e primeiro reforço, e cobertura completa da vacina contra poliomielite em nascidos entre 2017 e 2018, por estrato socioeconômico e municípios (capital e interior), Nordeste do Brasil (n = 12.137)

Uma proporção de 8,1% das crianças não apresentava nenhuma dose aplicada de vacina contra poliomielite, e 11,0% não completou o esquema vacinal.

A análise de regressão múltipla destacou as seguintes variáveis ​​associadas à vacinação incompleta contra a poliomielite: não ser beneficiário do PBF (OR-a 1,15, IC_95%_ 1,01;1,29), renda familiar de até R$ 1.000,00 (OR-a 1,19, IC_95%_ 1,06;1,35), mãe sem trabalho remunerado (OR-a 1,21, IC_95%_ 1,08;1,35), mais de um filho por mãe (OR-a 1,16, IC_95%_ 1,11;1,21), não ter cartão de vacinação (OR-a 18,06, IC_95%_ 10,01;32,61) e utilizar serviço privado de vacinação (OR-a 1,46, IC_95%_ 1,23;1,74) (Tabela 3).

**Tabela 3:** Razão de chances (*odds ratio*, OR) bruta e ajustada e intervalo de confiança de 95% (IC_95%_ ), acerca da associação de fatores sociodemográficos com a vacina ção incompleta contra poliomielite, em nascidos entre 2017 e 2018, residentes em capitais e municípios do interior, Nordeste do Brasil, 2020-2022 (n = 12.137)

Características	OR bruta(IC_95%_)	p-valor	OR ajustada(IC_95%_)	p-valor
Estrato socioeconômico				
A	1,66 (1,46;1,89)	0,260	–	–
B	1,40 (1,23;1,59)		–	
C	1,00		–	
D	1,23 (1,08;1,4)		–	
**Município**				
Capital	1,00	0,679	–	–
Interior	1,03 (0,91;1,16)		–	
**Da família**				
**Programa Bolsa Família**				
Sim	1,00	< 0,001	1,00	0,161
Não	1,20 (1,10;1,32)		1,15 (1,01;1,29)	
**Renda familiar mensal (R$)**				
≤ 1.000,00	1,12 (1,00;1,25)	< 0,001	1,19 (1,06;1,35)	0,143
1.001,00-3.000,00	1,00		1,00	
3.001,00-8.000,00	1,12 (0,97;1,29)		0,94 (0,79;1,12)	
≥ 8.001,00	1,74 (1,47;2,05)		1,21 (0,97;1,51)	
**Maternas**				
**Faixa etária ao nascimento da criança (anos)**				
< 20	1,00	0,243	–	–
20-34	1,30 (0,97;1,74)		–	
≥ 35	1,33 (0,99;1,79)		–	
**Escolaridade (anos)**				
0-8	1,00	0,003	1.00	0,941
9-12	1,04 (0,88;1,23)		1,13 (0,94;1,36)	
13-15	0,91 (0,79;1,06)		1,04 (0,87;1,24)	
≥ 16 anos	1,29 (1,10;1,51)		1,16 (0,93;1,47)	
**Trabalho remunerado**				
Sim	1,00	< 0,001	1,00	0,003
Não	1,29 (1,18;1,41)		1,21 (1,08;1,35)	
**Número de filhos vivos** **(média)**				
Média de mais de um filho por mãe	1,10 (1,07;1,14)	< 0,001	1,16 (1,11;1,21)	< 0,001
**Da criança**				
**Sexo**				
Masculino	1,00	0,995	–	–
Feminino	1,00 (0,91;1,09)		–	
**Possui caderneta de vacinação**				
Sim	1,00	< 0,001	1,00	< 0,001
Não	13,01 (8,19;20,66)		18.06 (10.01;32.61)	
**Uso de serviço privado para a vacinação**				
Sim	1,69 (1,50;1,90)	< 0,001	1.46 (1.23;1.74)	< 0,001
Não	1,00		1,00	
**Frequenta creche/escola**				
Sim	1,00	< 0,001	1,00	0,051
Não	1,19 (1,09;1,31)		1,11 (1,00;1,23)	

O estrato A apresentou maior proporção de crianças sem registro de doses aplicadas (14,7%, IC_95%_ 9,5;22,0) e maior proporção de crianças com esquema incompleto de doses (19,0%, IC_95%_ 13,6;25,9). Entre as capitais, Natal foi a que apresentou a maior proporção de crianças sem registro de doses (13,3%, IC_95%_ 7,6;22,1). Para as doses incompletas, destacaram-se Natal (20,5%, IC_95%_ 14,0;29,1), João Pessoa (17,3%, IC_95%_ 13,7;21,7) e Recife (16,1%, IC_95%_ 11,2;22,4). Nos municípios do interior, Vitória da Conquista teve maior proporção de crianças sem registro de nenhuma dose aplicada da vacina da poliomielite (14,5%, IC_95%_ 6,3;29,8) (Tabela 4).

**Table:** Não vacinação, vacinação incompleta, cobertura da vacina contra a poliomielite e taxa de abandono (%) e intervalo de confiança de 95% (IC_95_), em nascidos entre 2017 e 2018, por estrato socioeconômico, capitais e municípios de interior, Nordeste do Brasil, 2020-2022 (n = 12.137)

Variáveis	Cobertura vacinal			Taxa de abandono		
	Não vacinação	Vacinação incompleta	Vacinação completa	Segunda dose	Terceira dose	Primeiro reforço
	% (IC^95%^)	% (IC^95%^)	% (IC^95%^)	%	%	%
Estratos socioeconômicos						
A	14,7 (9,5;22,0)	19,0 (13,6;25,9)	66,3 (58,3;73,5)	2,4	12,1	19,2
B	9,5 (6,3;14,1)	9,3 (6,8;12,6)	81,2 (76,0;85,5)	0,9	3,9	9,3
C	6,3 (4,8;8,4)	7,3 (5,9;8,9)	86,4 (84,1;88,5)	0,8	2,6	6,4
D	6,2 (4,0;9,3)	10,4 (8,7;12,3)	83,5 (80,2;86,4)	1,3	3,4	10,0
**Capitais**						
São Luís	4,0 (2,0;7,8)	10,3 (6,8;15,3)	85,7 (79,1;90,4)	0,7	2,0	10,5
Teresina	1,9 (1,1;3,4)	6,9 (4,3;10,9)	91,2 (86,7;94,3)	1,9	4,4	7,0
Fortaleza	11,6 (7,8;17,0)	10,7 (7,1;16,0)	77,6 (72,3;82,3)	3,8	6,1	11,0
Natal	13,3 (7,6;22,1)	20,5 (14,0;29,1)	66,2 (56,2;74,9)	1,8	14,7	22,7
João Pessoa	9,6 (4,9;18,0)	17,3 (13,7;21,7)	73,1 (66,3;79,0)	0,0	4,9	16,4
Recife	7,6 (3,8;14,4)	16,1 (11,2;22,4)	76,4 (66,3;84,2)	-2,0	6,0	14,4
Maceió	12,8 (4,9;29,6)	8,4 (5,4;12,9)	78,8 (66,3;87,5)	0,4	2,1	8,3
Aracaju	11,3 (6,4;19,1)	6,6 (4,2;10,2)	82,1 (74,4;87,8)	2,7	2,3	5,4
Salvador	3,8 (2,4;6,0)	8,7 (6,2;12,1)	87,5 (83,0;91,0)	1,4	2,6	7,6
Capitais	8,2 (6,4;10,4)	11,4 (9,8;13,2)	80,4 (77,7;82,9)	1,4	4,8	10,9
**Interior**						
Imperatriz	3,7 (1,9;6,9)	9,8 (6,9;13,6)	86,5 (81,6;90,3)	0,6	1,9	8,2
Sobral	7,8 (2,6;21,0)	9,9 (6,1;15,7)	82,3 (69,4;90,4)	0,2	7,3	8,6
Caruaru	2,8 (1,1;6,7)	6,3 (3,6;10,7)	91,0 (85,5;94,5)	0,1	2,5	5,2
Vitória da Conquista	14,5 (6,3;29,8)	4,0 (2,2;7,4)	81,5 (66,6;90,7)	1,4	2,7	4,5
Interior	7,2 (4,3;11,7)	7,2 (5,5;9,3)	85,7 (81,1;89,3)	0,6	3,2	6,4
**Geral**	8,1 (6,5;10,1)	11,0 (9,6;12,7)	80,9 (78,4;83,1)	1,3	4,6	10,5

A taxa de abandono foi maior no 1º reforço (10,5%), quando comparada à primeira dose, com menor redução para a terceira (4,6%) em relação à primeira dose. O estrato A apresentou as maiores taxas de abandono para o 1º reforço (20,0%) e terceira dose (12,1%) em relação à primeira, sendo mais expressiva em Natal (abandono de 27,1% no primeiro reforço em relação à primeira dose; e de 14,5% da terceira, em relação à primeira dose) e em Imperatriz (abandono de 10,0% do primeiro reforço em relação à primeira dose, e de 2,7% da terceira em relação à primeira dose) (Tabela 4).

## DISCUSSÃO

Evidenciou-se, neste estudo, situação crítica da cobertura das vacinas contra poliomielite em crianças com até 24 meses de idade, em capitais e municípios do interior do Nordeste do Brasil. Ressalta-se que não houve alcance da meta de 95% para o esquema vacinal proposto pelo PNI entre nascidos vivos em 2017-2018. De forma crítica, quase um quinto da população infantil analisada não tinha vacinação completa para as vacinas da poliomielite, e – o que é mais preocupante –, 8,4% das crianças neste estudo (mais de mil crianças) não tinham registro de nenhuma dose de vacina contra essa doença imunoprevenível potencialmente grave.

A não vacinação e a vacinação incompleta contra a poliomielite esteve associada em conjunto a fatores que refletem parte das iniquidades sociais entre as crianças com baixas coberturas vacina^is^.^14^

A combinação desse contexto de subvacinação no Brasil à fragilização das ações de vigilância da circulação de poliovírus selvagem, oriundo de áreas persistentemente endêmicas no mundo, e de PVDV, pelo uso contínuo da VOPb, além d**e** fluxos migratóri^os^,^15^ ampliam o risco para reintrodução dessa doença, ameaça real e crítica pela possibilidade de Emergência de Saúde Pública de Importância Internacional (ESPI^I).2,16^,^17^

Nesse aspecto, o êxito no controle da poliomielite requer a incorporação de estratégias de imunização com foco no fornecimento de vacinas de qualidade e implementação de campanhas, com amplo envolvimento governamental, científico e comunitário, por ações de microplanejamento, com atuação direta no território sob monitoramen^to.15,18^,^19^

Quedas expressivas das coberturas vacinais ocorreram em todas as regiões do Bras^i^l,^8^ com tendência decrescente continuada na última década, de forma mais expressiva após 2015-20^16^.^20^ Esse fato foi amplificado durante a pandemia de covid-19 pela elevada taxa de abandono e heterogeneidade das coberturas vacina^is,21^,^22^ elevando o risco de reintrodução da poliomielite e de outras doenças imunopreveníve^is.15,18^,^19^ Nesse contexto, são exigidas melhorias sustentadas na cobertura vacinal e no acesso equitativo à vacinação, principalmente para recuperação e superação dos níveis anteriores à pandem^ia^.^23^

Chama a atenção, neste estudo, o declínio do alcance da meta proposta pelo PNI, quando comparada às coberturas das doses iniciais e sequenciais do esquema da vacina da poliomielite. Nesse patamar, o Brasil situa-se como país de altíssimo ri^s^co^8^ para reintrodução da poliomielite, tendo as piores situações registradas nos municípios de Natal (capital) e Vitória da Conquista (interio^r)^.^12^

Considerando-se a extrema gravidade dessa doença e os contextos de vulnerabilidade vivenciados na região Nordeste do Brasil, um chamamento urgente à rápida e assertiva intervenção, com forte participação de governos, comunidade científica e população, deve ser considerado prioritár^i^o.^8^

A não disponibilidade da caderneta de vacinação pela mãe/responsável, durante a ida à unidade de saúde para vacinação da criança, demonstrou ser fator operacional associado à cobertura vacinal incompleta contra poliomieli^te^.^12^ Ressalta-se que essa e outras barreiras de acesso às ações de imunização requerem novas estratégias para alcance de cobertura vacinal dentro das metas nacionais e internaciona^is.1,^ 2,8

Além da maior vulnerabilidade social atrelada a menores coberturas para vacinas contra poliomielite, verificou-se também que ser residente em áreas social e economicamente mais favoráveis esteve associado à não vacinação, circunstância também evidenciada em outros estudos no Brasil, considerando-se populações com maior acesso à intern^et.^ 21 Discute-se se o maior acesso a redes sociais contendo informações inadequadas, forte propagação de *fake news* e divulgação de discurso antivac^in^a^24^ teriam potencializado a não vacinação. Outros estudos no país têm demonstrado heterogeneidade, com melhor alcance da cobertura vacinal em áreas de menor vulnerabilidade soci^al^.^20^

Nesse mesmo contexto, crianças com menores coberturas das vacinas da poliomielite residentes em estratos socioeconômicos mais favoráveis das capitais brasileiras e municípios do interior pesquisadas tiveram maior frequência de vacinação no serviço priva^do.^ 25 De forma inversa, crianças/famílias que utilizam o serviço público de saúde apresentaram maior cobertura vacinal, provavelmente em decorrência d**o** vínculo estabelecido com a atenção primária à saúde (APS), e, dessa forma, manutenção de seguimento do calendário vacinal atualiza^do.25^,^26^ A interface deficiente entre rede privada e serviço público de saúde, em ações de rotina e campanhas nacionais de multivacinação, bem como o não encaminhamento de dados de doses aplicad^as^,^25^ são aspectos que podem ter contribuído e devem ser revisitados.

O PBF traz em perspectiva a necessidade de seguimento adequado à vacinação, uma de suas condicionalidades. Em maior proporção em pais/responsáveis do estrato socioeconômico menos favorável, o programa foi evidenciado como política governamental relevante para adesão à vacinação, e, assim, para alcance de cobertura adequada das vacinas contra a poliomieli^te^.^27^

Baixa renda, ausência de trabalho e mais de um filho por mãe são questões que refletem a desigualdade social, sendo apontados em diferentes estados da região Nordeste como fatores associados à não vacinação, e trazem em perspectiva contextos que amplificam limitações nas condições de saúde, especialmente na população infant^il^,^25^ por restrição a direitos sociais básic^os^.^13^

O iminente e constante risco da reintrodução da poliomielite no Brasil, em decorrência do atual cenário epidemiológico e social, indica a necessidade de ações contundentes para o fortalecimento no SUS de programas de vacinaçã**o** no Nordes^te.28^,^29^

Para tanto, há necessidade de ampliação de estratégias mais efetivas, considerando o rastreamento de crianças não vacinadas para a manutenção de elevada e homogênea cobertura vaci^nal15^,^16^ – principais barreiras de acesso à vacinação –, maior disponibilidade de serviços de vacinação e profissionais de saúde qualificados para acolhimento e redução de perdas de oportunidad^es.12,25^,^30^ Há uma clara necessidade de se estruturarem processos de microplanejamento nos territórios da APS, para melhor compreensão dos fatores envolvid^os^,^19^ além de se garantir decisão polít^i^ca^8^ para a implementação de medidas para alcance, de forma homogênea, da cobertura vacinal contra poliomielite no Nordes^te^.^25^

Destaca-se a relevância do profissional de saúde vinculado às ações de vacina^ç^ão^6^ e da necessidade de fortalecimento da formação e do apoio técnico à operacionalização das ações para efetivação de estratégias de vacinação adequadas e eficazes, diante do contexto da situação de saúde loc^al^.^19^

Registra-se ainda a necessidade de fortalecimento das ações de vigilância. Todos os países devem garantir alta cobertura vacinal contra a poliomielite em sua população, para alcance da meta de erradicação global, com intervenções apropriadas a sistemas nacionais de saúde. Para além das ações de rotina a serem intensificadas, a vigilância ambiental e de águas residuais deve ser implementada, para permitir a detecção precoce da transmissão “silenciosa” do poliovírus na população, avançando em ações centradas exclusivamente na vigilância clínica da paralisia flácida aguda apenas na definição de ca^s^o.^2^

As limitações desta pesquisa estão relacionadas ao método estabelecido para a utilização de dados do censo demográfico de 2010, com vistas à estratificação de setores censitários e composição da amostra. Na pesquisa de campo, as limitações deram-se em virtude das dificuldades de acesso às famílias, em decorrência da pandemia de covid-19, e da restrição do acesso de pesquisadores aos domicílios, principalmente nos estratos socioeconômicos mais elevados. Os resultados podem ter sido influenciados também pela limitada qualidade da imagem da caderneta de vacinação e pela ausência de padronização de registros de doses e vacinas aplicadas pelos serviços públicos e privados de vacinação. 

Como conclusão, este estudo traz evidência adicional acerca das baixas coberturas das vacinas contra a poliomielite, e do elevado percentual de não vacinação contra a poliomielite em crianças até 24 meses de idade residentes em capitais e municípios de grande porte populacional do interior do Nordeste. Esses achados sinalizam para o elevado risco de reintrodução da poliomielite, grave doença imunoprevenível, fornecendo suporte à tomada de decisão no planejamento das ações de vacinação. Recomenda-se a ampliação do acesso na APS, com a adoção de estratégias para alcance de adequadas coberturas para a poliomielite na população infantil do Nordeste brasileiro.
